# Hand Mortar for Mixing Amalgam

**Published:** 1885-01

**Authors:** D. Genese


ARTICLE XI.

### HAND MORTAR FOR MIXING AMALGAM.

BY D. GENESE, D. D. S.

The repeated expressions of prominent dentists that amalgam is better mixed in the hand, the warmth and plasticity helping the amalgamation, have induced me to offer the profession a Hand Mortar made of fine rubber, without a metal pigment, whereby the conditions most favorable to mixing may be maintained, at the same time preventing the hand from being stained, the absorption of mercury through the thick skin and the perspiration mixed with the metal causing spots. It may be manipulated with a glass pestle, or the finger covered with a rubber stall.

